# Impact of a Brief Group Intervention to Enhance Parenting and the Home Learning Environment for Children Aged 6–36 Months: a Cluster Randomised Controlled Trial

**DOI:** 10.1007/s11121-017-0753-9

**Published:** 2017-01-20

**Authors:** N. J. Hackworth, D. Berthelsen, J. Matthews, E. M. Westrupp, W. Cann, O. C. Ukoumunne, S. K. Bennetts, T. Phan, A. Scicluna, M. Trajanovska, M. Yu, J. M. Nicholson

**Affiliations:** 1 Parenting Research Centre, Level 5, 232 Victoria Parade, East Melbourne, 3002 Victoria Australia; 2 Judith Lumley Centre, La Trobe University, Level 3, 215 Franklin St., Melbourne, 3000 Victoria Australia; 3 Murdoch Childrens Research Institute, 50 Flemington Rd, Parkville, 3052 Victoria Australia; 4 School of Early Childhood, Queensland University of Technology, Level 4, B Block, Victoria Park Road, Kelvin Grove, 4059 Queensland Australia; 5 National Institute for Health Research Collaboration for Leadership in Applied Health Research and Care, South West Peninsula (PenCLAHRC), University of Exeter, Exeter, EX1 2LU UK; 6 Department of Paediatrics, Faculty of Medicine, Dentistry and Health Sciences, The University of Melbourne, Parkville, 3052 Victoria Australia; 7 Australian Institute of Family Studies, 485 La Trobe St., Melbourne, 3000 Victoria Australia

**Keywords:** Early childhood intervention, Home learning environment, Cluster randomised controlled trial, Parenting, Parent–child interactions

## Abstract

This study evaluated the effectiveness of a group parenting intervention designed to strengthen the home learning environment of children from disadvantaged families. Two cluster randomised controlled superiority trials were conducted in parallel and delivered within existing services: a 6-week parenting group (51 locations randomised; 986 parents) for parents of infants (aged 6–12 months), and a 10-week facilitated playgroup (58 locations randomised; 1200 parents) for parents of toddlers (aged 12–36 months). Each trial had three conditions: intervention (*smalltalk group*-*only*); enhanced intervention with home coaching (*smalltalk plus*); and ‘standard’/usual practice controls. Parent-report and observational measures were collected at baseline, 12 and 32 weeks follow-up. Primary outcomes were parent verbal responsivity and home learning activities at 32 weeks. In the infant trial, there were no differences by trial arm for the primary outcomes at 32 weeks. In the toddler trial at 32-weeks, participants in the *smalltalk group*-*only* trial showed improvement compared to the standard program for parent verbal responsivity (effect size (ES) = 0.16; 95% CI 0.01, 0.36) and home learning activities (ES = 0.17; 95% CI 0.01, 0.38) but *smalltalk plus* did not. For the secondary outcomes in the infant trial, several initial differences favouring *smalltalk plus* were evident at 12 weeks, but not maintained to 32 weeks. For the toddler trial, differences in secondary outcomes favouring *smalltalk plus* were evident at 12 weeks and maintained to 32 weeks. These trials provide some evidence of the benefits of a parenting intervention focused on the home learning environment for parents of toddlers but not infants. Trial Registration: 8 September 2011; ACTRN12611000965909.

**Electronic supplementary material:**

The online version of this article (doi:10.1007/s11121-017-0753-9) contains supplementary material, which is available to authorized users.

Policy attention is increasingly focused on early childhood intervention to ensure that all children commence school with the skills required for successful academic participation (Council of Economic Advisors 2014). From a young age, children raised in socially or economically disadvantaged families are at higher risk for poor cognitive, language, and socio-emotional development (Fernald et al. 2013; Nicholson et al. 2012). While early childhood initiatives to foster the development of young children from disadvantaged families can provide long-term societal benefits (Heckman et al. 2010), there is limited evidence on how to achieve this within existing services. The current research addresses this gap by evaluating the effectiveness of a parenting program, *smalltalk*, delivered within two Australian early childhood service sectors (Nicholson et al. 2016).

Socio-economic inequalities in children’s development are evident from a young age. By the time children commence school, those from more disadvantaged backgrounds lag behind their peers on the language, learning and socio-emotional skills they require for academic success (Nicholson et al. 2012). These early differences are maintained or widen with age, resulting in substantially poorer life-course outcomes (Kaplan et al. 2001; Poulton et al. 2002). Several modifiable characteristics of early childhood that contribute to inequalities in child development have been identified, offering the opportunity for early preventive interventions. Central to this are children’s early and repeated exposure to a home environment in which parents have a direct impact on the level and rate of children’s linguistic, cognitive and social/emotional development (Dreyer et al. 1996; Landry et al. 2006). In particular, parent–child interactions have been shown to play an important role in the link between socio-economic disadvantage and child development (Bradley et al. 2001; Miller et al. 2014; Yeung et al. 2002).

## Role of the Home Learning Environment in Supporting Children’s Development

In homes that are rich in literacy resources and where adults converse and read to children regularly, young children develop cognitive, communicative and social skills earlier than children whose homes do not provide such opportunities (Farver et al. 2006; Landry et al. 2006; Tamis‐LeMonda et al. 2004). Specific parenting behaviours associated with the development of these skills include warm, sensitive and responsive interactions, and engagement in cognitively stimulating activities (Landry et al. 2006; Tamis‐LeMonda et al. 2004). For example, parental warmth and expressions of affection and respect promote the acquisition of children’s sense of mastery, autonomy and self-efficacy. Parent interactions that respond to and build on a child’s interests result in sustained engagement and attention and create optimal conditions for learning (Farrant and Zubrick 2012). Parent verbal responsiveness during these interactions, increases children’s verbal expressions and their exposure to complex language (Hoff 2006). Frequent shared book reading provides an interactive context for acquiring and practicing verbal and conceptual skills. The poorer development of children from socially and economically disadvantaged families has been attributed, at least in part, to the relative lack of these behaviours (Miller et al. 2014; Raviv et al. 2004), highlighting an opportunity for early intervention and the prevention of developmental disparities.

## Enhancing the Home Learning Environment Through a Parenting Intervention

Early childhood services offer a range of supports to the families of young children. Individualised, intensive home visiting in the prenatal and infancy period is a well-established approach for enhancing the early home environment (Miller 2015; Olds 2002; Peacock et al. 2014). Home visiting is typically provided to highly vulnerable families and has achieved demonstrable reductions in child abuse and neglect, developmental impairment and antisocial behaviours (Olds 2002), with significant projected long-term societal benefits (Miller 2015). While an important component of early childhood services, this approach is resource intensive. It requires skilled, well-supported staff to ensure program integrity and fidelity and considerable variability in effectiveness has been documented (Peacock et al. 2014). Within any comprehensive system of services for families, challenges still remain on how to reach and engage the large number of families with developmentally vulnerable children who may benefit from additional support.

Group-based programs offer a feasible way of providing parenting support to large numbers of families and have been shown to be effective at changing the parenting skills associated with child behaviour problems (Sanders and Kirby 2014). Few have sought to change the quality of the home learning environment per se and evidence for the long-term effectiveness of group programs to be able to affect such change is lacking (Barlow et al. 2010; Center on the Developing Child at Harvard University 2007). It is also unclear whether group programs alone are sufficient to produce lasting change for more vulnerable families. This has led to recommendations for the use of hybrid delivery models that combine structured group parenting programs with a modified form of home visiting (Gomby 2007).

## The Present Study

The current study was commissioned by the Victorian State Government in Australia to design and evaluate a brief parenting intervention (*smalltalk*) to enhance the home learning environment of young children from disadvantaged families. Based on the developmental literature and applying a family-centred approach that built upon existing strengths, the program sought to increase quality parent–child interactions (responsive interactions characterised by parental sensitivity and warmth) and build a home environment rich in language and age-appropriate play activities. To maximise the potential for future wide-scale implementation and to ensure ecological and social validity, the program was designed for delivery within existing government-funded community services. In Australia, free, universal early childhood programs are provided to disadvantaged parents through two service sectors: the maternal and child health service and facilitated playgroups. The *smalltalk* program was tailored to the parents of infants in the maternal and child health service (the infant trial) and parents of toddlers in the facilitated playgroup service (the toddler trial). Program content was delivered at two levels of intensity: the group program alone (*smalltalk group*-*only*) and a hybrid approach combining the group program with individual home visits (*smalltalk plus*).

The aim of this study was to conduct two cluster randomised controlled trials, one in each service sector, to compare outcomes of the *smalltalk group*-*only* and *smalltalk plus* interventions with *standard care*. A mixed methods evaluation was developed guided by the smalltalk program logic model (see supplemental Fig. 1). Program inputs (e.g. resources and supports) and processes (e.g. program quality and participant engagement) were assessed via administrative records, staff training evaluations and practitioner ratings completed at the end of each program session. The focus of this paper is the key proximal outcomes targeted by the intervention, namely parenting behaviours and the home environment. We selected one primary outcome in each of these domains, parent verbal responsivity and home learning activities, assessed by parent-report five months after program completion (*T* = 32 weeks). Secondary outcomes included other measures of parenting behaviours (parent-reported warmth and irritability; directly observed parent–child interactions) and the home environment (parent-reported home literacy environment; household chaos). Distal parent and child outcomes were also assessed (e.g. parent wellbeing, community connectedness, child development) but are not reported here.

We hypothesised for both trials that parents allocated to the *smalltalk group*-*only* and *smalltalk plus* interventions would show greater improvements in the primary and secondary outcomes compared to parents allocated to the *standard* (control) programs. In the absence of prior evidence, we made no hypotheses regarding differences between the infant versus toddler services.

## Method

### Study Design

Two cluster randomised controlled trials were conducted in parallel, in the maternal and child health (infant trial) and facilitated playgroup (toddler trial) services. Ten local government authorities (LGAs) participated in each trial, with no LGA involved in both. LGAs nominated up to six community locations (clusters) for program delivery which were randomly allocated to one of three trial arms: *smalltalk group*-*only*, *smalltalk plus* or *standard*. Participants received the intervention offered by the location providing services to their geographic area. Outcomes were assessed at baseline, 12-week and 32-week follow-up. Study design and intervention details have been reported elsewhere (Nicholson et al. 2016).

### Randomisation

Allocation of locations was stratified by LGA using block randomisation. Locations were allocated in the order that they were consented, in batches using fixed block sizes of a multiple of 3 to maintain allocation concealment. Randomisation was performed by OU who was unaware of the identities of the locations and played no role in recruitment.

### Participants

Parents were recruited by LGA staff. Eligibility criteria included living within the geographical boundaries of a trial location; having at least one child in the age range for the offered program (6–12 months for the infant trial and 12–36 months for the toddler trial); and at least one indicator of social disadvantage including low family income, receipt of government benefits, single, socially isolated or young parent (≤25 years) and culturally or linguistically diverse background. Parents were not eligible if they were aged less than 18 years, did not speak English or were receiving intensive support or child protection services.

### The Intervention

The *smalltalk* programs were developed in collaboration with service providers and parents to maximise fit to existing service structures and workforce capabilities and to ensure acceptability and relevance to target families. Program content targeted behaviours that have been consistently associated with enhanced child language, communication and socio-emotional development (e.g. Kaiser and Hancock 2003; Landry et al. 2006). Specifically, it aimed to increase the frequency of five responsive parenting behaviours (tuning in, following the child’s lead, listening and talking, teachable moments and warm and gentle engagement) and five strategies for providing a stimulating home learning environment (shared book reading, supporting children’s play, learning through everyday routines, using community resources and monitoring use of media). Information was provided about three additional factors that have indirect effects on children, namely the importance of looking after oneself (self-care), having confidence in one’s parenting (personal agency) and building connections with other parents and services (community connectedness).

To support parent behaviour change, program delivery processes were structured to create a teaching and learning context based in families’ everyday routines and activities. Staff who facilitated groups or provided home coaching received training in how to support parents to engage effectively with their children using family-centred practices that sought to build parental agency and self-regulation (Campbell and Sawyer 2007; Sanders and Mazzucchelli 2013; Woods et al. 2011). Specific intervention strategies employed in the groups and during home visits included coaching, live and video modelling, opportunities for practice and feedback, supported by written resources and DVD models of the strategies in use.

Program structure and timing were tailored to the service delivery contexts. For the infant trial, the program was conducted as a parent education group in six 2-hour weekly sessions run through the maternal and child health service. Group facilitators discussed the parenting strategies, guided practice in the group and assisted parents to plan and review their use of the strategies at home. For the toddler trial, the program was provided in ten 2-hour weekly facilitated playgroup sessions for parents and children which were run over four terms per year. Group facilitators identified parents who were new to the playgroup at the start of each term. Over the ensuing 10 weeks, facilitators discussed the parenting strategies with these parents, structured play activities to enable practice and assisted parents to plan and review their use of the strategies at home. At the end of the 10 weeks, parents could remain in the playgroup, but were no longer targeted for discussion and practice of the strategies.

In both trials, parents in the *smalltalk plus* condition also received six fortnightly 1-hour visits from a home coach. Sessions reinforced the content covered in group sessions using a narrated DVD which guided the coach and parent through practice of the key parenting strategies (with modelling and video-feedback), planning and reviewing their use.

In the standard condition, no *smalltalk* program content was provided. Parents of infants received six weekly group sessions focusing on age-relevant parenting issues (e.g. feeding, sleeping, safety, exercise and behaviour). Parents of toddlers received ten weekly playgroup sessions conducted according to the guidelines for government-funded playgroups.

#### Program Staff

Group and home coaching sessions were delivered by 114 early childhood staff employed by the participating LGAs. Staff were aged 23 to 59 years (mean = 42) with between 0 and 37 years (mean = 15.5) experience in the early childhood community sector. All but one were female. Half (56%) had vocational qualifications (e.g. diploma or certificate), 28% held a bachelor degree and 14% held post-graduate degrees. Qualifications were predominantly in the fields of community services (46%), education (29%) and health (12%). Staff received 2 or 3 days (depending on role) training from the research team in program content and processes. To avoid cross-contamination, staff were only trained in one intervention condition. Self-ratings completed after training (*n* = 109) indicated the majority believed they were ‘well-prepared’ and ‘confident’ and anticipated ‘little difficulty’ implementing the program (Hackworth et al. 2013).

#### Quality and Fidelity

Additional strategies employed to promote the quality and fidelity of program delivery included provision of a comprehensive program manual for staff, and written resources that included wall posters, conversation cards and activity, session planning and tip sheets. Process data collection required staff to plan sessions in advance, record and review the content delivered and rate the quality of each session. For home coaching sessions, the narrated DVD ensured fidelity and consistent quality. As per usual practice within each service, facilitators and home coaches were provided with regular supervision by coordinators within their organisation (usually weekly) who were trained in all elements of program delivery. Support around program delivery was available from the research team via phone or email, and program coordinators in each LGA received approximately weekly telephone calls to support implementation.

### Measures

#### Data Collection

Parents provided informed consent for data collection. At baseline, 12 weeks and 32 weeks, they completed a 20–30 minute computer-assisted telephone interview (CATI) and a video-recorded parent–child interaction at home. Parents were reimbursed with gift cards ($50 for full assessment; $20 for partial assessment) and a book for their child at each time point. Staff who conducted the in-home video-recording of parent–child interactions were not blind to participants’ trial arm or data collection time point. Staff employed to conduct the CATIs or code the observation data were independent of the study team and blind to the participants’ trial arm status. Coders were also blind to the data collection time point.

#### Parent-Report Measures

Parent verbal responsivity to their child was assessed on the 4-item Parental Verbal Responsivity subscale of the StimQ-T (Dreyer et al. 1996). Items (e.g. ‘How often do you play finger/rhyming games with your child?’) were rated from 1 = *not at all* to 4 = *every day*.

Parental warmth was assessed on a 6-item scale from the Longitudinal Study of Australian Children (LSAC; Zubrick et al. 2014). Items (e.g. ‘How often do you hug or hold your child for no reason?’) were rated from 1 = *never*/*almost never* to 5 = *always*/*almost always*.

Parental irritability was assessed using a 5-item scale from LSAC (Zubrick et al. 2014). Items (e.g. ‘In the last 4 weeks how often have you lost your temper with your child?’) were rated from 1 = *not at all* to 5 = *all the time*.

Home learning activities were assessed using the 5-item LSAC modification of the Early Childhood Longitudinal Study, Kindergarten Cohort measure (National Center for Education Statistics 2002). Items (e.g., ‘How often do you tell stories to your child?’) were rated from 1 = *not at all* to 4 = *every day*.

Home literacy environment was assessed with 6 items deemed most relevant to the literacy modelling behaviours addressed in the program (e.g. ‘How often do you read to your child?’) selected from the 15-item Home Literacy Environment Index (Griffin and Morrison 1997). Items were rated 0 to 1 or 0 to 2 with higher scores indicating a more positive literacy environment (possible total score 0 to 11).

Household chaos was assessed with the 6-item short-form of the Confusion, Hubbub, and Order Scale (CHAOS) measuring order and routine in the home (Johnson, Martin, Brooks-Gunn, and Petrill 2008). Items (e.g., ‘Has a regular bedtime routine’) were rated *yes*/*no*, then summed and dichotomised to ‘any chaos’ (score > 0) or ‘no chaos’.

Higher total scores on parent-reported measures indicated higher levels of the construct. Internal reliability was acceptable for warmth and irritability (Cronbach’s alphas ranged from 0.68 to 0.76), and modest for verbal responsivity (0.45 infant trial; 0.49 toddler trial). Internal reliability was not computed for activity checklists or dichotomised indicators.

#### Observational Measures

The Indicator of Parent–Child Interaction (Baggett and Carta 2006) observation protocol was used to assess parent–child interactions that promote positive child cognitive, language and social-emotional skills. This involved direct observation of parent–child interactions during a set of standardised tasks: free play (4 min); looking at books (2 min); dressing (2 min); and a distraction task (2 min; for children over 12 months). Interactions were video-recorded and coded for the frequency of six behaviours in two domains: ‘parent facilitators’ (warmth and acceptance; descriptive language; follows child’s lead; maintains child’s interest) and ‘parent interrupters’ (harsh comments; restrictions). Inspection of distributions on IPCI sub-scales showed that interrupter behaviours were rare and not suitable for analysis. Coding using a standardised protocol was undertaken by two independent, accredited, post-graduate research assistants at the University of Kansas under the supervision of the research scientist who developed the method [KB]. Accreditation involved a half-day training with sample assessments scored until criterion were consistently reached (i.e. 80% agreement with a gold-standard rater). The IPCI has been reported to have strong psychometric properties, with evidence for concurrent, discriminant and criterion validity (Greenwood et al. 2008). In the current study, inter-rater agreement on 20% of observations independently coded was 87.4%, which is consistent with previously reported figures (Baggett and Carta 2006).

### Procedure

All LGAs in the state of Victoria (*N* = 79) were invited to express interest in study participation (see Nicholson et al. 2016). Twenty LGAs (10 in each of the infant and toddler trials) consented and were retained for the full trial duration. Each nominated up to six locations randomised to deliver *smalltalk plus*, *smalltalk group*-*only* or *standard* programs.

### Sample Size Calculation

Our target was to recruit 22 locations (clusters) and 308 parent–child dyads (14 per location) in each trial arm for each RCT. The intended sample size is large enough to detect a difference of 0.3 standard deviation units (effect size) between any two trial arms with 90% power at the 5% level of significance, allowing for an intra-cluster (intra-location) correlation coefficient of 0.01 and 15% participant attrition at follow-up.

### Statistical Methods

Data were analysed using Stata, version 13.1. Baseline characteristics were summarised by trial arm status and compared for parents included in the analyses versus those excluded due to missing data. An ‘available cases’ approach was used at each time point for parent-report data. For example, for pre to post analyses, data were only included if the participant had both pre and post data for outcomes and adjustment variables. To address the primary and secondary research aims, outcomes for the *smalltalk group*-*only* and *smalltalk plus* trial arms were compared to the standard arm at 12 and 32 weeks using the intention-to-treat principle with participants analysed according to their trial arm. Continuous outcomes were compared using random effects (‘multilevel’) linear regression models and binary outcomes were compared using marginal logistic regression models using generalised estimating equations with information sandwich (‘robust’) estimates of standard error, specifying an exchangeable correlation structure. These methods allow for the correlation of responses from the same cluster. Baseline characteristics accounted for in adjusted analyses were child age and gender, single parent family status, language other than English spoken at home, young mother (≤25 years), low education (<year 12), no parent employed and baseline scores for the outcome measure. Effect sizes for continuous outcomes were calculated by dividing the adjusted mean difference between trial arms by the standard deviation in the control arm.

## Results

**Fig. 1:**
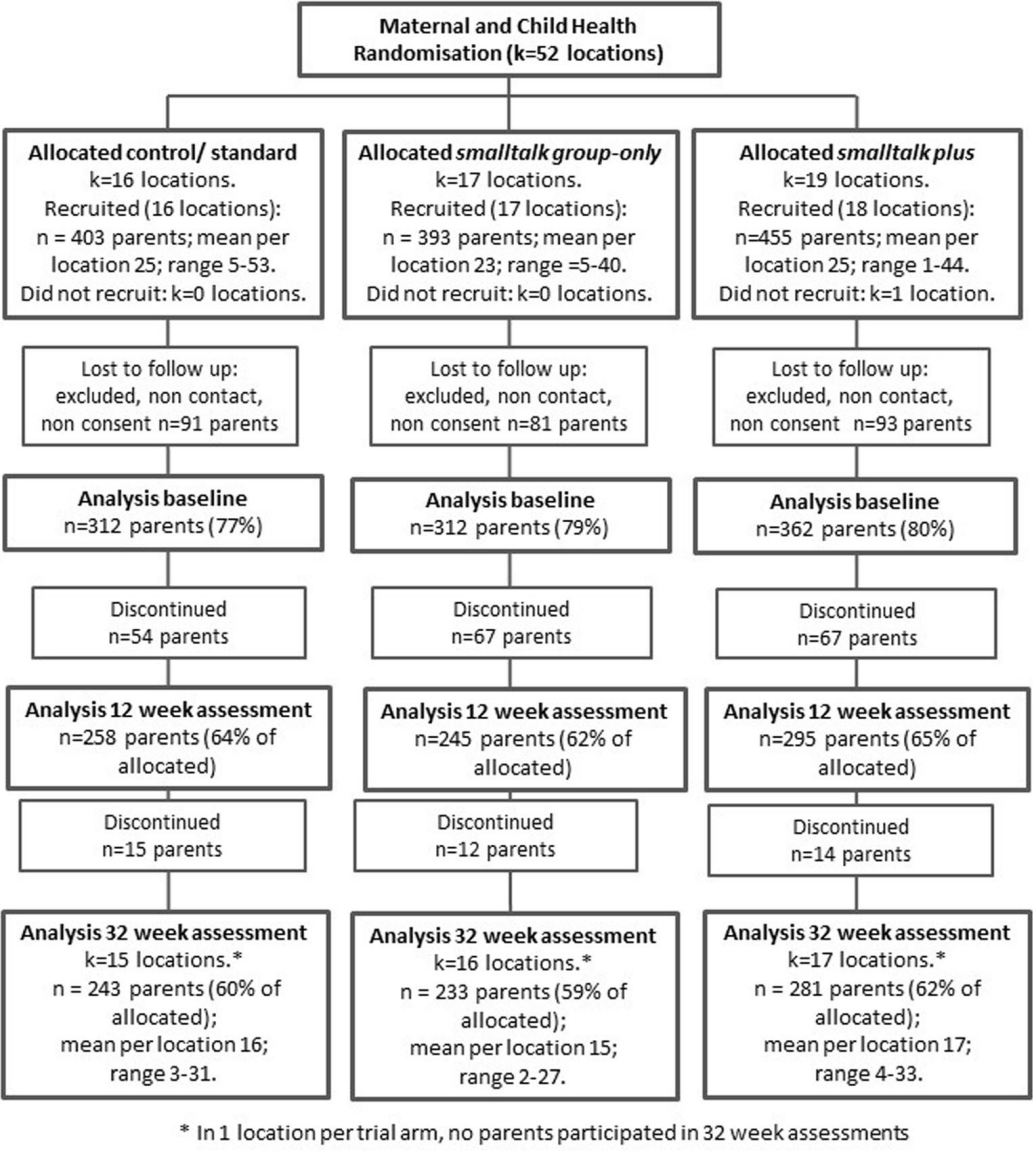
Participants flow for the infant trial (Nicholson et al. 2016)

**Fig. 2:**
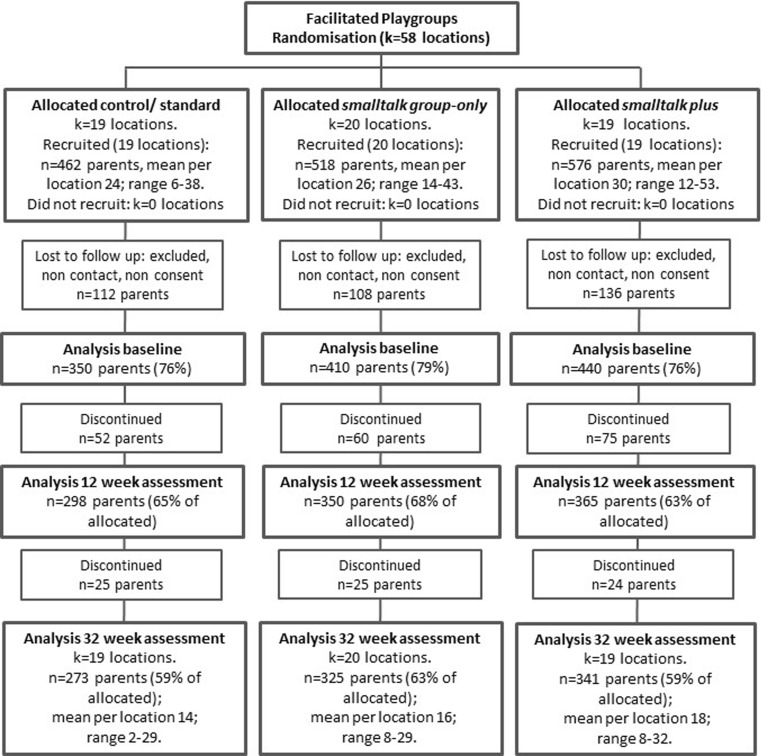
Participants flow for the toddler trial (Nicholson et al. 2016)

Participant flow for each trial is shown in Figs. 1 and 2. In the infant trial, baseline data were collected from 986 parents across 51 locations (clusters), of whom 81% (*n* = 798) and 77% (*n* = 757) were retained to 12 and 32 weeks follow-up. In the toddler trial, baseline data were collected from 1200 parents across 58 locations of whom 84% (*n* = 1013) and 78% (*n* = 939) were retained to 12 and 32 weeks, respectively. Due to the high costs of coding, around 20% of participants with video data at two or more time points were randomly selected (stratified by location) for coding. Final samples varied due to inability to code some observations (e.g. poor video quality; non-English words spoken).

Participant characteristics for the whole sample are summarised in Supplemental materials, Table 1. The mean (SD) age of children was 8.0 (2.3) months in the infant trial and 22.3 (7.2) months in the toddler trial. Compared to parents with complete data, those with missing follow-up data (*n* = 181 and 170 in the infant and toddler trials, respectively) were more likely to be young (infant 35 vs. 15%; toddler 22 vs. 7%), single parents (infant 19 vs. 11%; toddler 20 vs. 10%), on low income (infant 33 vs. 19%; toddler 36 vs. 19%), receiving government benefits (infant 33 vs. 16%; toddler 29 vs. 16%), with low education (infant 29 vs. 12%; toddler 21 vs. 10%) or with no employed parent in the home (infant 24 vs. 12%; toddler 26 vs. 11%). There were no differences for language spoken at home or country of birth.

Groups were run successively by school term so that each location ran four groups per year. A total of 160 parent groups (infant trial) and 224 playgroups (toddler trial) were provided. In both trials, the average group size was 6 parents which was lower than intended (infant programs designed for 6–8 parents; toddler programs designed for 10–15 parents). The average proportion of sessions attended was similar across trials and trial arms, ranging from 59 to 64%. In *smalltalk plus*, participants received the majority of planned home coaching visits (infants 81%; toddlers 78%) with 67% receiving all six visits.

Intra-cluster correlation coefficients from the regression models for parent-reported measures at 12 and 32 weeks ranged from 0 to 0.05. Tables 1 (infant trial) and 2 (toddler trial) present unadjusted and adjusted comparisons of the two active intervention conditions against the standard condition.

**Table 1:** Infant sample parent-reported and observed outcomes: Unadjusted and adjusted comparisons at 12 and 32 week follow-up

	*Smalltalk group*-*only* vs standard				*Smalltalk plus* vs standard			
	Mean difference				Mean difference			
	Unadj	Adjusted (95% CI)	*pa *	ESa	Unadj	Adjusted (95% CI)	*pa *	ESa
Parent report, 12-week assessment (*N* = 798)								
Parent verbal responsivity	0.04	0.14 (−0.14, 0.42)	0.34	0.08	0.22	0.35 (0.08, 0.61)	0.01	0.18
Parenting warmth	−0.20	−0.19 (−0.57, 0.19)	0.33	0.09	0.28	0.23 (−0.14, 0.60)	0.22	0.11
Parenting irritability	0.22	0.28 (−0.03, 0.59)	0.07	0.12	−0.14	−0.06 (−0.35, 0.24)	0.70	0.03
Home learning activities	0.19	0.14 (−0.19, 0.48)	0.41	0.06	0.45	0.46 (0.14, 0.78)	0.005	0.20
Home literacy environment	0.36	0.20 (−0.07, 0.47)	0.14	0.11	0.36	0.35 (0.09, 0.61)	0.007	0.19
High household chaos (OR)	1.07	1.07 (0.64, 1.78)	0.81		0.94	0.80 (0.43, 1.48)	0.48	
Observed, 12-week assessment (*N* = 100)								
Acceptance and warmth	0.27	4.69 (−5.21, 14.60)	0.35	0.23	−2.76	−0.98 (−11.50, 9.55)	0.86	−0.05
Descriptive language	3.41	5.81 (−5.76, 17.37)	0.33	0.24	18.16	15.38 (3.13, 27.63)	0.01	0.63
Follow child’s lead	9.41	9.78 (1.07, 18.50)	0.03	0.50	11.23	10.99 (1.60, 20.38)	0.02	0.56
Maintains child’s interest	0.67	0.86 (−6.69, 8.41)	0.82	0.05	1.23	0.49 (−7.65, 8.62)	0.91	0.03
Parent report, 32-week assessment (*N* = 757)								
Parent verbal responsivity	0.03	0.10 (−0.20, 0.39)	0.52	0.05	0.07	0.16 (−0.13, 0.44)	0.29	0.08
Parenting warmth	−0.23	−0.23 (−0.57, 0.10)	0.17	0.12	0.11	0.03 (−0.29, 0.35)	0.86	0.01
Parenting irritability	0.32	0.41 (0.06, 0.75)	0.02	0.18	0.22	0.30 (−0.03, 0.62)	0.08	0.13
Home learning activities	−0.03	−0.12 (−0.44, 0.20)	0.48	0.06	0.03	0.01 (−0.30, 0.31)	0.97	0.00
Home literacy environment	0.17	0.01 (−0.26, 0.28)	0.95	0.00	0.003	0.06 (−0.20, 0.31)	0.67	0.03
High household chaos (OR)	1.13	1.25 (0.72, 2.17)	0.43		1.08	1.09 (0.56, 2.12)	0.81	
Observed, 32-week assessment (*N* = 88)								
Acceptance and warmth	−0.31	4.88 (−5.21, 14.97)	0.34	0.20	−0.80	4.99 (−6.12, 16.09)	0.38	0.20
Descriptive language	6.26	6.61 (−7.91, 21.12)	0.37	0.28	5.10	3.78 (−11.82, 19.38)	0.64	0.16
Follow child’s lead	4.68	5.50 (−5.87, 16.86)	0.34	0.31	2.73	4.43 (−7.93, 16.78)	0.48	0.25
Maintains child’s interest	−0.20	0.73 (−6.41, 7.86)	0.84	0.06	1.97	3.66 (−4.21, 11.52)	0.36	0.32

Adjusted models included the outcome measure at baseline and the following baseline covariates: child age, child gender, single parent, language other than English spoken at home, mother ≤25 years of age, mother did not complete year 12 and no parent employed

For parent-reported data: at 12-week assessment, *N* = 258 standard; *N* = 245 *smalltalk group*-*only*; *N* = 295 *smalltalk plus*. At 32-week assessment, *N* = 243 standard; *N* = 233 *smalltalk group*-*only*; *N* = 281 *smalltalk plus*. Samples were reduced due to missing data by 1–3 participants on each measure at 12 weeks and by 1 participant each on verbal responsivity and home learning activities at 32 weeks. For observational data: at 12-week assessment, *N* = 35 standard; *N* = 34 *smalltalk group*-*only*; *N* = 31 *smalltalk plus*. At 32-week assessment, *N* = 29 standard; *N* = 32 *smalltalk group*-*only*; *N* = 27 *smalltalk plus*

^a^Refers to adjusted values

**Table 2:** Toddler sample parent-reported and observed outcomes: Unadjusted and adjusted comparisons at 12 and 32 week follow-up

	*Smalltalk group*-*only* vs standard				*Smalltalk plus* vs standard			
	Mean difference				Mean difference			
	Unadj	Adjusted (95% CI)	*pa *	ESa	Unadj	Adjusted (95% CI)	*pa *	ESa
Parent report, 12-week assessment (*N* = 1013)								
Parent verbal responsivity	−0.02	0.11 (−0.17, 0.39)	0.45	0.05	−0.06	0.17 (−0.11, 0.45)	0.23	0.08
Parenting warmth	−0.04	0.02 (−0.37, 0.41)	0.93	0.01	−0.25	−0.08 (−0.48, 0.31)	0.68	0.04
Parenting irritability	−0.02	−0.13 (−0.47, 0.20)	0.43	0.04	0.08	−0.29 (−0.62, 0.04)	0.08	0.10
Home learning activities	0.12	0.22 (−0.05, 0.49)	0.11	0.10	0.24	0.35 (0.08, 0.61)	0.01	0.16
Home literacy environment	−0.14	−0.05 (−0.29, 0.19)	0.70	0.02	0.01	−0.09 (−0.33, 0.15)	0.44	0.05
High household chaos (OR)	1.02	0.95 (0.66, 1.38)	0.80		1.13	1.04 (0.68, 1.60)	0.86	
Observed, 12 week assessment (*N* = 119)								
Acceptance and warmth	−0.79	−0.44 (−8.96, 8.08)	0.92	0.02	1.44	5.32 (−4.70, 15.34)	0.30	0.20
Descriptive language	9.73	7.87 (−0.51, 16.25)	0.07	0.35	22.33	17.35 (7.84, 26.86)	<0.001	0.77
Follow child’s lead	−3.61	−3.44 (−12.61, 5.72)	0.46	0.15	5.81	4.08 (−6.29, 14.45)	0.44	0.18
Maintains child’s interest	5.04	2.02 (−6.66, 10.71)	0.65	0.09	12.33	11.27 (1.16, 21.37)	0.03	0.52
Parent report, 32-week assessment (*N* = 939)								
Parent verbal responsivity	0.24	0.35 (0.02, 0.69)	0.04	0.16	−0.10	0.08 (−0.25, 0.42)	0.63	0.04
Parenting warmth	0.12	0.09 (−0.26, 0.43)	0.62	0.04	−0.01	0.11 (−0.23, 0.45)	0.53	0.05
Parenting irritability	−0.25	−0.33 (−0.72, 0.05)	0.09	0.11	0.05	−0.28 (−0.66, 0.10)	0.15	0.09
Home learning activities	0.30	0.39 (0.03, 0.74)	0.03	0.17	0.10	0.26 (−0.10, 0.62)	0.15	0.11
Home literacy environment	0.001	0.004 (−0.24, 0.24)	0.97	0.00	0.26	0.11 (−0.13, 0.34)	0.38	0.05
High household chaos (OR)	1.15	1.22 (0.77, 1.91)	0.40		1.36	1.38 (0.88, 2.18)	0.16	
Observed, 32-week assessment (*N* = 128)								
Acceptance and warmth	−1.88	−1.07 (−9.94, 7.79)	0.81	0.04	0.55	5.35 (−4.58, 15.29)	0.29	0.19
Descriptive language	3.54	2.97 (−5.07, 11.01)	0.47	0.14	12.95	9.44 (0.53, 18.35)	0.04	0.46
Follow child’s lead	4.03	1.91 (−5.59, 9.41)	0.62	0.09	11.86	7.62 (−0.65, 15.89)	0.07	0.35
Maintains child’s interest	1.86	−0.59 (−8.77, 7.59)	0.89	0.03	13.68	12.23 (3.16, 21.29)	0.008	0.55

Adjusted models included the outcome measures at the baseline and the following baseline covariates: child age, child gender, single parent, language other than English spoken at home, mother ≤25 years of age, mother did not complete year 12 and no parent employed

For parent-reported data: at 12-week assessment, *N* = 298 standard; *N* = 350 *smalltalk group*-*only*; *N* = 365 *smalltalk plus*. At 32-week assessment, *N* = 272 standard; *N* = 325 *smalltalk group*-*only*; *N* = 342 *smalltalk plus*. Samples were reduced due to missing data by 1–2 participants on each measure at 12 weeks and at 32 weeks. For observational data: at 12-week assessment, *N* = 37 standard; *N* = 54 *smalltalk group*-*only*; *N* = 28 *smalltalk plus*. At 32-week assessment, *N* = 40 standard; *N* = 53 *smalltalk group*-*only*; *N* = 35 *smalltalk plus*

^a^Refers to adjusted values

### Infant Trial

On the parent-reported measures at 12 weeks follow-up, parents allocated to *smalltalk group*-*only* did not show statistically significantly greater improvements on any outcomes compared to parents allocated to the standard condition. Parents allocated to *smalltalk plus* showed greater improvements compared to parents in the standard arm on verbal responsivity (ES = 0.18; 95% CI 0.04, 0.32), home learning activities (ES = 0.20; 95% CI 0.07, 0.41) and the home literacy environment (ES = 0.19; 95% CI 0.05, 0.32).

On the observed measures of parent–child interactions at 12 weeks, parents allocated to *smalltalk group*-*only* showed greater improvements in following their child’s lead (ES = 0.50; 95% CI 0.05, 0.94) compared to standard. Parents allocated to *smalltalk plus* showed greater improvements in following their child’s lead (ES = 0.56; 95% CI 0.08, 1.03) and use of descriptive language (ES = 0.63; 95% CI 0.13, 1.13) compared to standard.

On the parent-reported measures at 32 weeks, one statistically significant difference was found. Parents allocated to *smalltalk group*-*only* showed a greater increase in irritability (ES = 0.18; 95% CI 0.03, 0.39) than the standard condition. On the observed measures at 32 weeks, no significant differences were found.

### Toddler Trial

On the parent-reported measures at 12 weeks follow-up, there were no differences for parents allocated to *smalltalk group*-*only* compared to standard. Parents allocated to *smalltalk plus* showed greater improvements in home learning activities (ES = 0.16; 95% CI 0.04, 0.32) compared to standard. On the observed measures at 12 weeks, there were no differences between parents allocated to *smalltalk group*-*only* and standard. Parents allocated to *smalltalk plus* showed greater improvement in use of descriptive language (ES = 0.77; 95% CI 0.35, 1.20) and maintaining their child’s interest (ES = 0.52; 95% CI 0.05, 0.98) compared to standard.

On the parent-reported measures at 32 weeks, parents allocated to *smalltalk group*-*only* showed greater improvements in verbal responsivity (ES = 0.16; 95% CI 0.01, 0.36) and home learning activities (ES = 0.17; 95% CI 0.01, 0.38) compared to standard. Parents allocated to *smalltalk plus* did not show greater improvements on any primary or secondary outcome measures. On the observed measures at 32 weeks, there were no statistically significant differences between *smalltalk group*-*only* and standard. Parents allocated to *smalltalk plus* showed greater improvement in use of descriptive language (ES = 0.46; 95% CI 0.03, 0.89) and maintaining their child’s interest (ES = 0.55; 95% CI 0.14, 0.96) compared to standard.

## Discussion

This study examined the effects on parenting and the home learning environment of a brief group intervention provided to the parents of infants through the maternal and child health service and to the parents of toddlers through facilitated playgroups. The study was successful in recruiting a large sample of disadvantaged parents, with relatively high rates of program attendance. In the infant trial, there was no support for our primary hypothesis of greater sustained improvements on parent-reported verbal responsivity or home learning activities for parents allocated to either *smalltalk* intervention compared to the standard condition. While greater improvements were seen on these measures at 12 weeks for parents allocated to the *smalltalk plus* condition, differences were not sustained to the 32-week follow-up. In the toddler trial, our primary hypothesis was supported for parents allocated to *smalltalk group*-*only* (with small effect sizes), but was not supported for parents allocated to *smalltalk plus*.

Evaluations of early home learning interventions face conceptual and methodological challenges. The outcomes of interest (parenting and the home learning environment) are complex processes not adequately captured through unidimensional measures (Zubrick et al. 2014). Additionally, the family-centred approach used in the *smalltalk* programs seeks to harness parents’ individual strengths and provide families with choice as to the specific skills targeted for development (Epley et al. 2010). To address this complexity, we measured a range of secondary outcomes reflecting other dimensions of parent–child interaction and the home learning environment. When looking beyond the primary outcomes, we see a somewhat different pattern of findings. In the infant trial, there continued to be little evidence of sustained effects. However, when the secondary outcomes were considered for the toddler trial, there was greatest evidence for effects at 32 weeks for parents allocated *smalltalk plus*, with moderate-to-large effect sizes on three observed measures.

The *smalltalk* programs were designed as a relatively ‘light touch’ intervention that could be delivered within the existing services without the need to employ specialist staff. The relatively modest findings reported here may accurately reflect the modest potential of this approach. Alternatively, it is possible that the intervention was not delivered well. Within the constraints of this large-scale and geographically distributed study, it was not possible to collect objective measures of program quality and fidelity. Steps that were taken to ensure quality delivery included the narrow focus on a set of ten parent behaviours; resources and activities co-designed by early childhood staff to ensure fit with their existing skills and work environments; use of a highly structured weekly program, supported by written and DVD materials for parents and staff to ensure consistency in the information presented to parents; and the collection of process data that required staff to record the session content and quality. As inconsistent delivery is a particular threat to the effectiveness of home visiting interventions (Peacock et al. 2014), the *smalltalk* home coaching sessions were led by a DVD ‘instructor’ who guided both the coach and parent through the session content.

A unique strength of the current study was the parallel RCTs conducted in the two service sectors where *smalltalk* was being considered for implementation. This provided comparable data collected under identical research conditions. The infant and toddler programs covered the same content, but differed in method of instruction and program duration. The infant program was shorter (6 week) and structured like a parent education group with the facilitator leading group discussions. The toddler playgroup program was longer (10 weeks), and the content was covered through incidental teaching methods. This required facilitators to structure play activities in a way that would elicit opportunities to discuss, model and practice the content. In this less-structured approach, there are greater risks of parents missing out on *smalltalk* content (due to poor structuring of activities), but conversely, greater flexibility to catch up on content missed due to parental absences. Parents could also continue playgroup attendance over the longer term, and while they were no longer targeted for *smalltalk* discussions, they continued to be exposed to an environment where this was occurring with other parents. Our results suggest that the more instructional parent education group approach available to parents of infants had some initial benefits that were not sustained. In contrast, the playgroup-based incidental teaching method available to the parents of toddlers was associated with few initial benefits, but moderate-to-large benefits 5 months after program completion, and had benefits across a greater range of outcome measures when paired with home coaching.

Differences between the infant and toddler *smalltalk* programs may also reflect differing parental perceptions about the relevance of the content to their current parenting demands. For the parents of infants, their child’s longer term development may be a less pressing concern than managing sleeping, feeding and adjusting to parenthood. However, a lack of perceived relevance seems unlikely given the consistently high participation (59–64% of group sessions; 78–81% of home coaching sessions) and study retention rates (75–79% retained to 32-week follow-up) achieved for both the infant and toddler programs. These rates are notable given the competing demands faced by parents of young children and the difficulties typically reported in recruiting and retaining disadvantaged families in interventions (Brown et al. 2012). For example, recent reviews indicate that 20 to 60% of those who enrol in home visiting programs drop out before completion (Gomby 2007; Peacock et al. 2014).

The current study was designed and conducted according to CONSORT guidelines for cluster randomised control trials (Campbell et al. 2012). Strengths include the location-based clustered design to minimise opportunities for cross-arm contamination between staff or parents, inclusion of the home coaching trial arm to explore whether an individual home-based component was necessary to effect change, comparison against an active control condition as a stringent test of whether any benefits exceeded those associated with programs currently available and comprehensive assessment of outcomes using both parent-reported and directly observed measures.

We also paid careful attention to the ecological and social validity of the *smalltalk* programs (Nicholson et al. 2016). Large-scale parenting interventions for disadvantaged families often report low uptake, high attrition and lack of parent engagement (Brown et al. 2012) and have mostly yielded modest results (Furstenberg 2011). Strong collaborative relationships with stakeholders from government and the early childhood service sector were maintained across all stages of the trial. The programs were co-produced with service end-users and parents to ensure that the methods of delivery were practical and achievable within existing structures and workforce competencies (ecological validity) and that the program had a high level of relevance, acceptability and usefulness to families (social validity). The intervention approach was well-received by staff, and our recruitment and retention rates indicate that the program was acceptable and engaging for parents.

This study had several limitations. First was the lack of objective measures of program quality and fidelity. While we developed administrative processes and program resources to maximise fidelity, it is likely that there was variation in staff competency, supervision and supports. Second, we could not afford to code observational data for all participants. Thirdly, we recruited 16 to 20 locations (clusters) per trial arm, which was short of the goal of 22 per arm, and it is possible that we were under-powered for some analyses.

## Conclusions and Implications

This is the first study in Australia and one of only a few internationally to examine the effectiveness of a group-based early home learning intervention, conducted as a rigorous cluster randomised controlled trial ‘in situ’. The *smalltalk* program was commissioned to address a gap in Australian early childhood services which currently provide universal well health checks and targeted intensive home visiting for highly vulnerable families. It was highly structured and accompanied by a suite of written and DVD materials to ensure existing staff were able to deliver the program competently. Study findings provide modest support for the benefits of the *smalltalk* approach as delivered via facilitated playgroups, with inconsistent results across the primary and secondary outcomes. On the basis of the two primary outcomes, there was evidence for small effects at 32 weeks for *smalltalk group*-*only* approach (compared to standard) and no effects on any secondary outcome measures. In contrast, the *smalltalk plus* approach showed no effects relative to standard programs on the primary outcomes, but moderate-to-large effects on two secondary outcomes. For the infant program, some early effects were evident, but these were not maintained to the 32 week follow-up.

Interventions that involve home visiting typically offer this as a stand-alone approach. In the current study, home coaching sessions sought to consolidate the skills learnt in the groups. While we found some evidence for the benefits of combining individual home coaching with the group program (on secondary but not primary outcomes), these do not appear sufficient to justify providing this component to all families. The home visiting component is costly in terms of the staffing required and further research is needed to determine whether some families require home coaching in order to achieve benefits. Identification of the level of intervention that is sufficient to promote change will allow the development of triage processes to ensure that home coaching can be effectively allocated to those who need it most.

In the context of a rigorous research trial, the *smalltalk* program has demonstrated its capacity to reach and retain the target sample, and to produce modest effects on parenting practices and the home learning environment. While promising, longer term assessment of child outcomes is required to determine whether this translates into improved developmental skills at school entry, with ongoing social and economic benefits. Additionally, even programs with strong evidence for efficacy often fail to produce the same effects on implementation and scale-up either because they do not continue to reach the target audience or to be delivered as intended. While the *smalltalk* program and processes have been designed to support successful scale-up, the degree to which program effects are maintained when *smalltalk* is taken to scale remains to be seen.

## Electronic Supplementary Material

Below is the link to the electronic supplementary material.

ESM 1 (JPG 88 kb)

ESM 2 (DOCX 37 kb)
