# Supplementary figures and images for: Impact of a Brief Group Intervention to Enhance Parenting and the Home Learning Environment for Children Aged 6–36 Months: a Cluster Randomised Controlled Trial
Source: Prev Sci. 2017 Jan 20;18(3):337–49. doi: 10.1007/s11121-017-0753-9 (PMC5352786; doi:10.1007/s11121-017-0753-9)

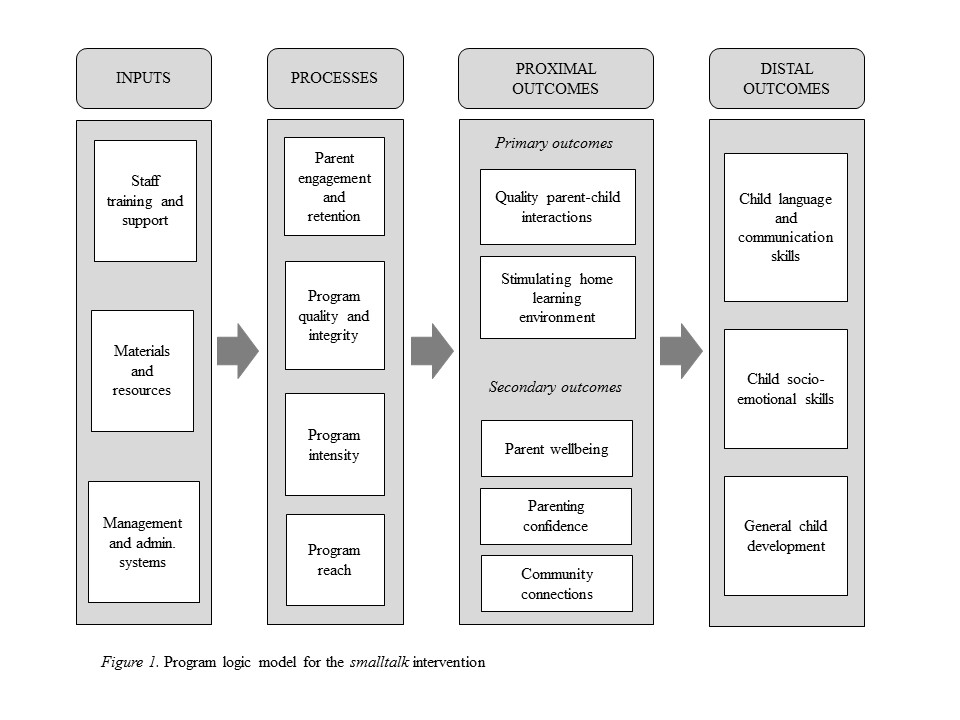

Supplement: Supplementary file 1 — (JPG 88 kb) [file 11121_2017_753_MOESM1_ESM.jpg]
