# Supplementary material for: Impact of a Brief Group Intervention to Enhance Parenting and the Home Learning Environment for Children Aged 6–36 Months: a Cluster Randomised Controlled Trial
Source: Prev Sci. 2017 Jan 20;18(3):337–49. doi: 10.1007/s11121-017-0753-9 (PMC5352786; doi:10.1007/s11121-017-0753-9)
Supplement: Supplementary file 2 — (DOCX 37 kb) [file 11121_2017_753_MOESM2_ESM.docx]

*Supplementary Table 1.* Participant characteristics at pre-assessment by condition and sample: means (standard deviation) or percentages.

|  | Infant Sample (N=986) | | | Toddler Sample (N=1200) | | | | |
| --- | --- | --- | --- | --- | --- | --- | --- | --- |
|  | standard | *smalltalk group-only* | *smalltalk plus* |  | standard | *smalltalk group-only* | *smalltalk plus* | |
| *Sample demographics* | N=312 | N=312 | N=362 |  | N=350 | N=410 | N=440 | |
| Child age in months | 7.9 (2.4) | 8.1 (2.2) | 8.0 (2.2) |  | 21.7 (7.5) | 22.4 (7.2) | 22.8 (7.1) |  |
| Male child | 47.4% | 53.9% | 49.7% |  | 51.7% | 48.5% | 45.7% |  |
| Child Indigenous Australian | 2.3% | 2.6% | 2.8% |  | 0.9% | 2.4% | 1.8% |  |
| Parents’ age ≤ 25 years | 19.2% | 18.3% | 19.3% |  | 9.7% | 9.5% | 9.3% |  |
| Single parent family | 12.5% | 13.1% | 12.4% |  | 13.7% | 9.3% | 11.3% |  |
| Parent born overseas | 16.0% | 12.2% | 13.3% |  | 34.9% | 31.3% | 31.0% |  |
| Language other than English | 13.1% | 10.9% | 13.8% |  | 34.3% | 35.7% | 29.4% |  |
| No parent employed | 10.3% | 15.1% | 16.0% |  | 13.4% | 12.4% | 14.6% |  |
| Parent did not complete year 12 | 13.1% | 15.1% | 15.8% |  | 12.0% | 11.5% | 11.3% |  |
| Income from pension/benefits | 16.1% | 21.5% | 19.1% |  | 19.7% | 15.9% | 17.4% |  |
| Annual income $36,400 or less | 19.3% | 22.8% | 21.5% |  | 23.8% | 20.4% | 21.1% |  |
| *Parent report measures* | N=312 | N=312 | N=362 |  | N=350 | N=410 | N=440 |  |
| Parent verbal responsivity | 13.3 (2.2) | 13.2 (2.3) | 13.2 (2.3) |  | 12.9 (2.4) | 12.9 (2.2) | 12.7 (2.5) |  |
| Parenting warmth | 28.4 (2.1) | 28.5 (2.0) | 28.5 (2.0) |  | 28.0 (2.3) | 27.9 (2.4) | 27.7 (2.6) |  |
| Parenting irritability | 7.7 (2.2) | 7.4 (2.1) | 7.6 (2.3) |  | 10.0 (3.0) | 10.1 (2.9) | 10.4 (3.0) |  |
| Home learning activities | 16.9 (2.5) | 17.0 (2.8) | 16.8 (2.8) |  | 17.2 (2.5) | 17.1 (2.6) | 17.0 (2.7) |  |
| Home literacy environment | 7.0 (1.9) | 7.2 (2.0) | 7.0 (2.0) |  | 7.1 (2.2) | 7.0 (2.2) | 7.2 (2.2) |  |
| High household chaos | 0.21 (0.40) | 0.20 (0.40) | 0.22 (0.42) |  | 0.25 (0.43) | 0.26 (0.44) | 0.26 (0.44) |  |
| *Observation measures* | n=38 | n=36 | n=34 |  | n=44 | n=58 | n=36 |  |
| Acceptance & warmth | 74.2 (20.2) | 68.4 (25.4) | 71.7 (24.9) |  | 63.7 (22.5) | 64.0 (23.8) | 56.4 (22.8) |  |
| Descriptive language | 53.0 (19.9) | 49.2 (24.1) | 51.4 (23.4) |  | 57.5 (22.6) | 60.9 (25.6) | 61.8 (23.6) |  |
| Follow child’s lead | 65.3(21.5) | 66.0 (18.9) | 65.8 (25.2) |  | 69.4 (21.7) | 71.3(21.6) | 74.4 (17.6) |  |
| Maintains child’s interest | 17.1 (21.8) | 13.8 (19.4) | 14.3 (15.8) |  | 20.6 (21.7) | 24.3 (26.5) | 21.9 (23.2) |  |
| *Note:* Samples were reduced due to missing data as follows: n=1 each for child Indigenous Australian, parents’ age ≤25, and single parent family; n=4 for income from pension/benefits; n=83 participants for annual income. Demographic data reproduced from [Nicholson et al. (2016)](#_ENREF_1). | | | | | | | |  |
